# Supplementary material for: Ab initio calculation of real solids via neural network ansatz
Source: Nat Commun. 2022 Dec 22;13:7895. doi: 10.1038/s41467-022-35627-1 (PMC9780243; doi:10.1038/s41467-022-35627-1)
Supplement: Supplementary file 1 — Supplementary information: Ab initio calculation of real solids via neural network ansatz [file 41467_2022_35627_MOESM1_ESM.pdf]

Supplementary information: Ab initio calculation of real solids via neural network ansatz

Xiang Li et al

Supplementary Note 1. Hyperparameters for simulations

The recommended hyperparameters are listed in Supplementary Table 1. Some employed hyperparameters of the presented results differ from the recommended ones, which are specially given in Supplementary Table 2.

Supplementary Table 1 | Recommended hyperparameters

Hyperparameter	Value	Hyperparameter	Value
Pretrain basis	cc-pVDZ	Pretrain iterations	1e3
Dimension of one electron layer **V**	256	Dimension of two electron layer **W**	32
Number of layers	4	Number of determinants	8
Optimizer	KFAC	Learning rate	3e-2
Damping	1e-3	Constrained norm of gradient	1e-3
Momentum of optimizer	0.0	Batch size	4096
Number of training steps	2e5	Clipping window of gradient	5
MCMC burn in	1e3	MCMC steps between each iterations	20
MCMC move width	2e-2	Target MCMC acceptance	55%
Precision	Float64	Number of inference steps	5e4

Supplementary Table 2 | Some system dependent hyperparameters

System	Layer dimension	Layer	Determinants	Batch size	Training steps	A100 cards	Wall time
Hydrogen chain	(256, 32)	3	8	4096	1e5	8	7 hours
Graphene	(256, 32)	4	8	4096	3e5	32	3 days
$2 \times 2 \times 2$ Lithium hydride	(256, 32)	4	8	4096	3e5	16	5 days
$3 \times 3 \times 3$ Lithium hydride	(256, 32)	4	1	8192	4e5	128	4 weeks
$2 \times 2 \times 2$ bcc-Lithium	(256, 32)	4	8	4096	2e5	16	1 week
14-electron HEG	(256, 32)	4	16	4096	2e5	8	1 day
54-electron HEG	(256, 32)	3	1	4096	3e5	32	2 days

Supplementary Note 2. Hydrogen chain

Supplementary Note 2.1 Training curve

The training curve of H_{10} in PBC is plotted in Supplementary Fig. 1. The correlation error is defined as

$$\text{Correlation error} = \left(1 - \frac{E_{\text{Net}} - E_{\text{HF}}}{E_{\text{DMC}} - E_{\text{HF}}}\right) \times 100\% \,, \tag{1}$$

where E_{HF} is calculated using a truncated cc-pVDZ basis set (exponent threshold = 0.1) and E_{DMC} is taken from Ref. [1].

Supplementary Note 2.2 H_{10} dissociation curve

Energy of H_{10} chain per atom is given in Supplementary Table 3. LR-DMC and VMC results are from Ref. [1], and HF results are also listed for comparison.

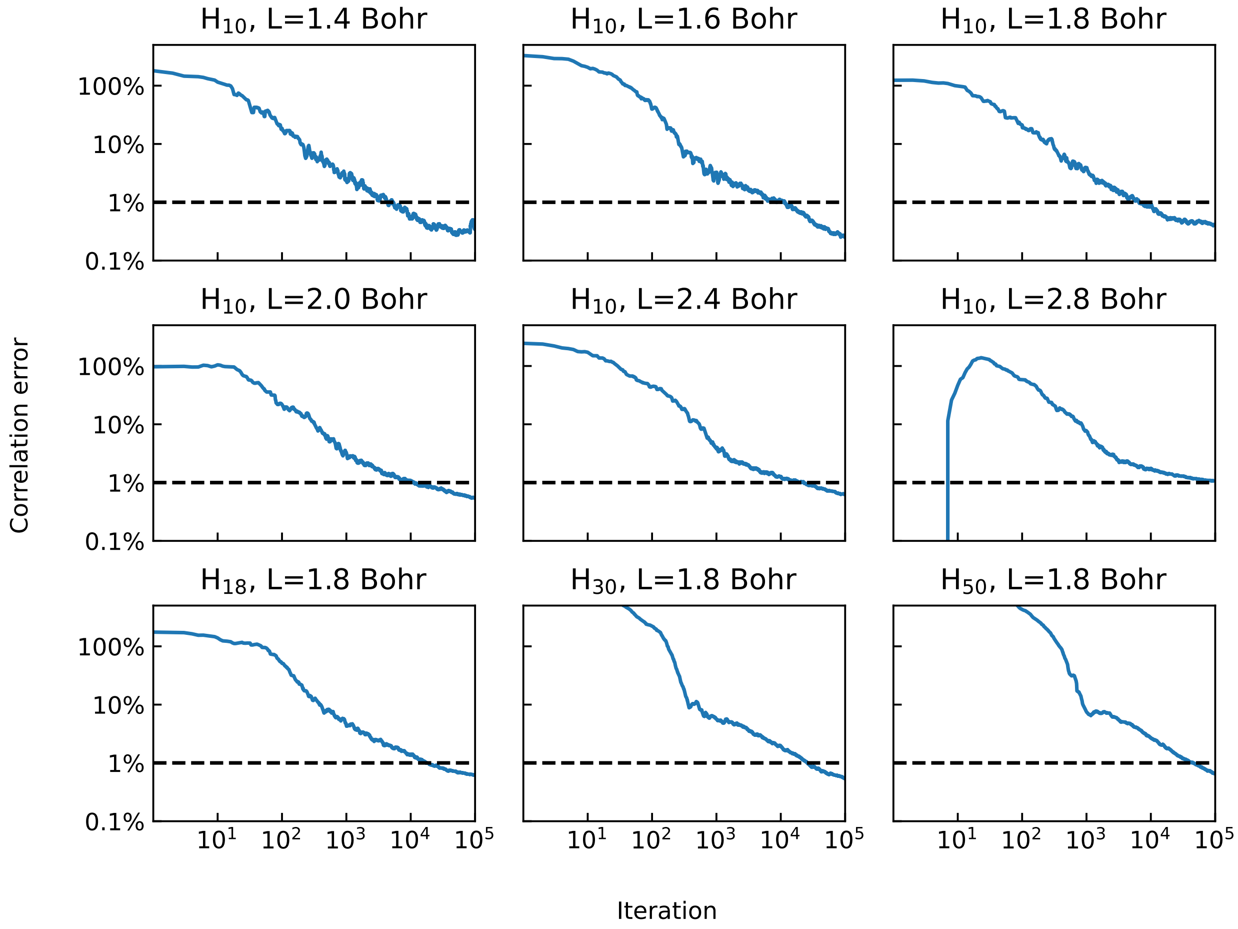

Supplementary Figure 1 | Hydrogen chain training curve. For clarity, at each iteration number, we plot the median correlation error of the last 10% of the corresponding iteration.

Supplementary Table 3 | Energy per atom of H_{10} chain. Energies are given in Hartree.

Bond length (Bohr)	Net	LR-DMC(LDA)	VMC(LDA)	HF
1.4	-0.551677(2)	-0.55178(1)	-0.55049(1)	-0.52058
1.6	-0.568740(1)	-0.56881(1)	-0.56752(1)	-0.53937
1.8	-0.572922(1)	-0.57304(1)	-0.57172(1)	-0.54425
2.0	-0.570401(1)	-0.57055(1)	-0.56911(1)	-0.54173
2.4	-0.556861(1)	-0.55703(1)	-0.55522(1)	-0.52690
2.8	-0.540783(1)	-0.54102(1)	-0.53831(1)	-0.50792

Supplementary Note 2.3 Finite-size error extrapolation

Energies of different hydrogen chains are given in Supplementary Table 4.

Supplementary Table 4 | Energies per atom of different hydrogen chains. Energies are given in Hartree and the bond length of hydrogen chain is fixed at 1.8 Bohr.

Size	Net	LR-DMC(LDA)	VMC(LDA)	HF
10	-0.572922(1)	-0.57304(1)	-0.57172(1)	-0.54425
18	-0.567776(1)	-0.56796(1)	-0.56644(1)	-0.53787
30	-0.566114(1)	-0.56627(1)	-0.56478(1)	-0.53581
50	-0.565419(1)	-0.56560(1)	-0.56409(1)	-0.53499

Supplementary Note 3. Graphene

Supplementary Note 3.1 Geometry

The primitive cell lattice vectors as well as carbon atom coordinates are given in Supplementary Table 5. The size of supercell is 2×2.

Supplementary Table 5 | Geometry of Graphene.

Atom	Position (Å)	Lattice vector	Position (Å)
C1	(1.421, 0.0, 0.0)	$\mathbf{a}_1$	(2.1315, -1.2306, 0.0)
C2	(2.842, 0.0, 0.0)	$\mathbf{a}_2$	(2.1315, 1.2306, 0.0)
		$\mathbf{a}_3$	(0, 0, 52.9177)

Supplementary Note 3.2 Twist average boundary condition (TABC)

A 3×3 Monkhorst-Pack mesh in the first Brillouin zone of the supercell reciprocal space with Γ point centered is used to approximate the twist average integral, which reads

$$E_{\text{TABC}} = \frac{\Omega_S}{(2\pi)^3} \int_{\text{1.B.Z.}} d^3\mathbf{k}_S \, \frac{\psi^*_{\mathbf{k}_S} \hat{H}_S \psi_{\mathbf{k}_S}}{\psi^*_{\mathbf{k}_S} \psi_{\mathbf{k}_S}} \approx \frac{1}{9} E_{\mathbf{k}_1} + \frac{2}{3} E_{\mathbf{k}_2} + \frac{2}{9} E_{\mathbf{k}_3},$$
$$\mathbf{k}_1 = 0, \ \mathbf{k}_2 = \frac{1}{3}\mathbf{b}^S_1 + \frac{1}{3}\mathbf{b}^S_2, \ \mathbf{k}_3 = \frac{2}{3}\mathbf{b}^S_1 + \frac{1}{3}\mathbf{b}^S_2, \tag{2}$$

and the weight factors origin from the different number of symmetry equivalent $\mathbf{k}$ points.

Supplementary Note 3.3 Training curves

Training curves at different $\mathbf{k}_S$ are plotted in Supplementary Fig. 2.

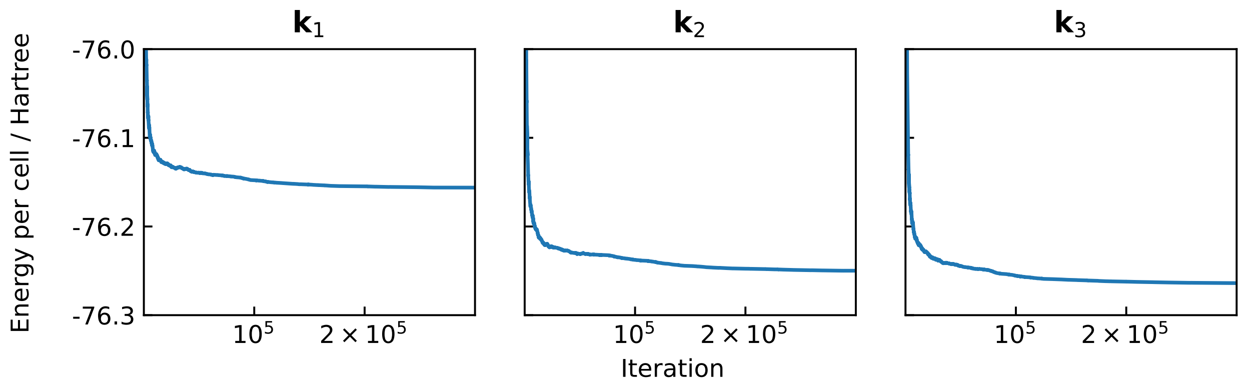

Supplementary Figure 2 | 2×2 Graphene training curve. For clarity, at each iteration number, we show the median energy per unit cell over the last 10% of iteration.

The final results are listed in Supplementary Table 6. The energy of an isolated carbon atom is taken from Ref. [2], $E = -37.84471$ Hartree.

Supplementary Table 6 | Energy per unit cell of graphene at different twists.

	$\mathbf{k}_1$	$\mathbf{k}_2$	$\mathbf{k}_3$
Net (Hartree)	-76.15588(6)	-76.24949(5)	-76.26314(5)
HF (Hartree)	-75.69786	-75.80015	-75.80983

Supplementary Note 3.4 Structure factor correction

TABC technique is usually combined with structure factor corrections [3], and the combination is now seen as the standard scheme of applying QMC to solids. Structure factor $S(\mathbf{k})$ is calculated to correct the exchange-correlation part, namely V_{xc}, of the total potential energy, which reads

$$\frac{\Delta V_{\mathrm{xc}}}{N_e} = \frac{2\pi}{\Omega_S} \lim_{\mathbf{k}\to 0} \frac{S(\mathbf{k})}{\mathbf{k}^2} ,$$
$$S(\mathbf{k}) = \frac{1}{N_e}[\langle \rho(\mathbf{k})\rho^*(\mathbf{k})\rangle - \langle \rho(\mathbf{k})\rangle\langle \rho^*(\mathbf{k})\rangle] , \; \rho(\mathbf{k}) = \sum_i \exp(i\mathbf{k}\cdot\mathbf{r}_i) , \tag{3}$$

where $\mathbf{r}_i$ refers to the coordinate of each electron, and N_{e} denotes the number of electrons in the simulation cell. The calculated $S(\mathbf{k})$ and corresponding ΔV_{xc} of Γ point is plotted in Supplementary Fig. 3, and corrections of all twists are quite close to each other.

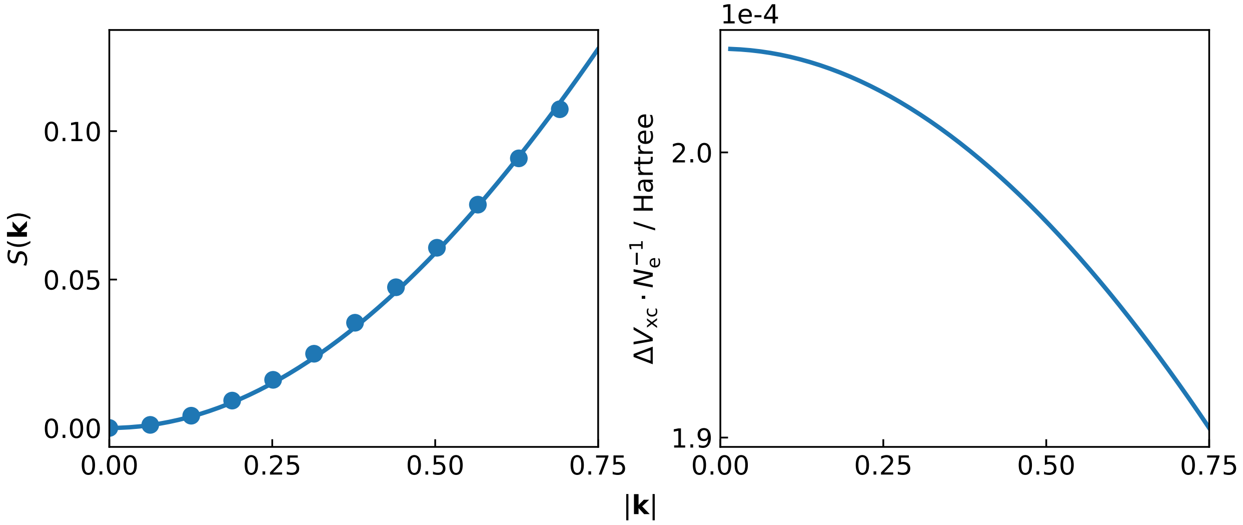

Supplementary Figure 3 | Structure factor correction of Graphene. The lines are fitted with the formula: $S(\mathbf{k}) = 1 - \exp(-a \cdot \mathbf{k}^2)$.

The final correction from structure factor is 0.00122 Hartree / atom.

Supplementary Note 4. Lithium hydride

Supplementary Note 4.1 Geometry

Lithium hydride crystal has a rock-salt structure, whose lattice vectors and atom positions are given in Supplementary Table 7.

Supplementary Table 7 | Geometry of LiH crystal

Atom	Position	lattice vector	Position
Li	(0.0, 0.0, 0.0)	$\mathbf{a}_1$	(0.0, L/2, L/2)
H	(L/2, L/2, L/2)	$\mathbf{a}_2$	(L/2, 0.0, L/2)
		$\mathbf{a}_3$	(L/2, L/2, 0.0)

Supplementary Note 4.2 Training curves

Training curves of the $2 \times 2 \times 2$ LiH crystal is plotted in Supplementary Fig. 4.

Supplementary Note 4.3 Dissociation curve

The energy of $2 \times 2 \times 2$ LiH is listed in Supplementary Table 8. The energy of an isolated lithium atom is taken from Ref. [2], $E = -7.47798$ Hartree. Corresponding Hatree-Fock corrections are calculated with the cc-pVDZ basis set and the convergence behavior of HF calculation is plotted in Supplementary Fig. 5.

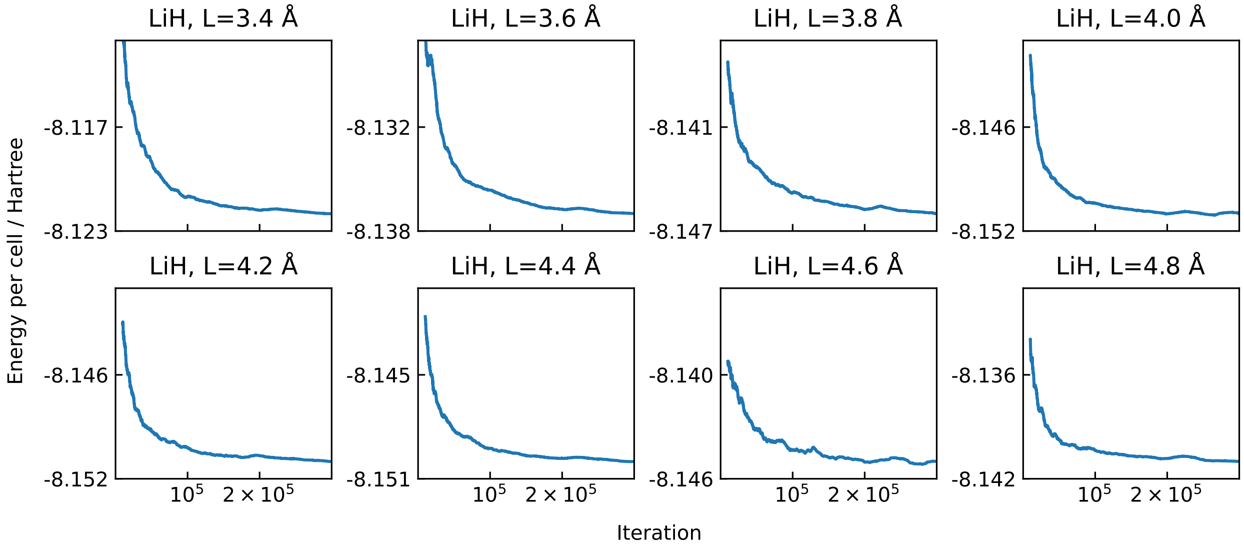

Supplementary Figure 4 | $2 \times 2 \times 2$ LiH training curve. For clarity, at each iteration number, we show the median energy of unit cell over the last 10% of iteration.

Supplementary Table 8 | Energy per unit cell of $2 \times 2 \times 2$ LiH crystal. Energies are all given in Hartree.

L (Å)	Net	HF	HF correction	L (Å)	Net	HF	HF correction
3.4	-8.12185(1)	-8.02622	-0.0099	4.2	-8.15112(1)	-8.06172	-0.0004
3.6	-8.13738(1)	-8.04396	-0.0067	4.4	-8.14967(1)	-8.06052	0.0009
3.8	-8.146147(1)	-8.05458	-0.0042	4.6	-8.14502(2)	-8.05712	0.0020
4.0	-8.15096(1)	-8.06006	-0.0021	4.8	-8.14094(1)	-8.05205	0.0030

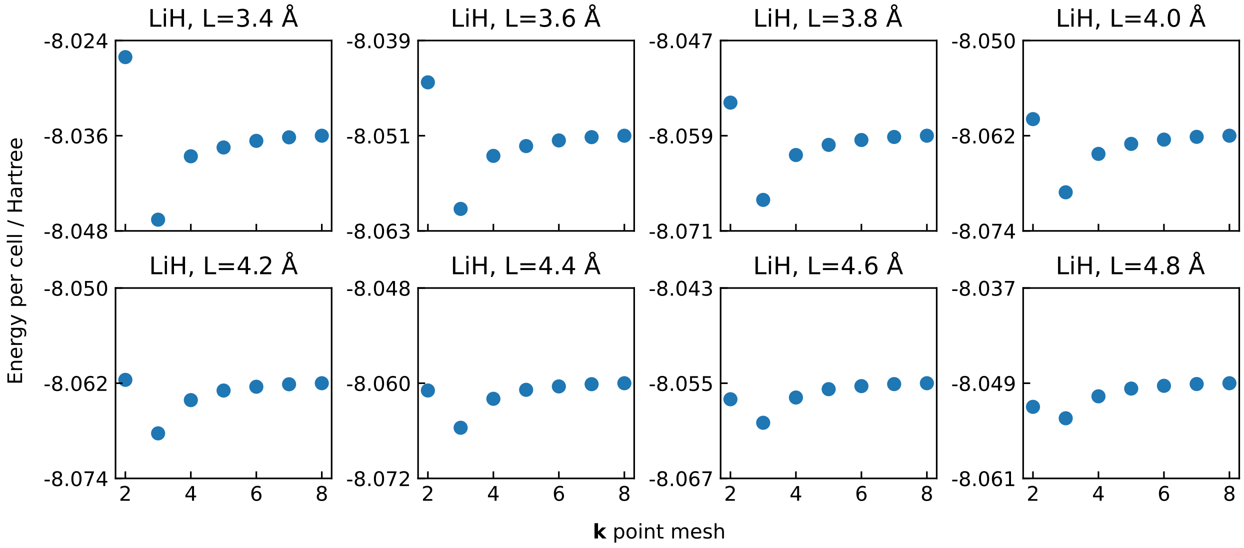

Supplementary Figure 5 | Hartree-Fock corrections. The convergence behavior of HF calculations with respect to the number of **k** points.

Supplementary Note 4.4 Birch-Murnaghan fit

The third order Birch-Murnaghan equation of state is employed to fit the dissociation curve, which reads

$$E(V) = E_0 + \frac{9V_0B_0}{16}\left\{\left[\left(\frac{V_0}{V}\right)^{2/3} - 1\right]^3 B_0' + \left[\left(\frac{V_0}{V}\right)^{2/3} - 1\right]^2\left[6 - 4\left(\frac{V_0}{V}\right)^{2/3}\right]\right\}, \tag{4}$$

where E_0, V_0, B_0, B_0' are fitted quantities, their results and corresponding experiment data [4] are listed in Supplementary Table 9.

Supplementary Table 9 | Parameters of Birch-Murnaghan equation of state

	a_0 (Å)	B_0 (GPa)	E_{coh} (eV)
Net	4.022	36.89	-4.757
Exp	4.061(1)	33-38	-4.778,-4.759

Supplementary Note 4.5 $3 \times 3 \times 3$ LiH

The training curve of the $3 \times 3 \times 3$ LiH crystal at its equilibrium lattice constant L = 4.061Å is plotted in Supplementary Fig. 6, corresponding Hartree-Fock corrections are also given. The final inference results from neural network are listed in Supplementary Table 10.

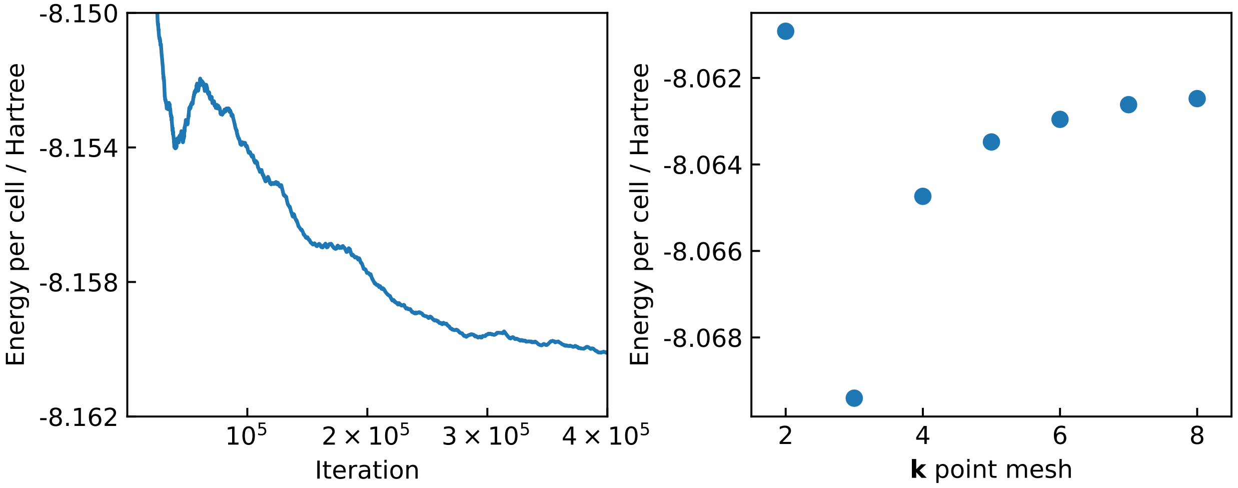

Supplementary Figure 6 | $3 \times 3 \times 3$ LiH. Left panel plots the training curve of the $3 \times 3 \times 3$ LiH. For clarity, at each iteration number, we show the median energy per unit cell over the last 10% of iteration. Right panel plots the corresponding Hartree-Fock corrections with the cc-pVDZ basis set.

Supplementary Table 10 | Energy of the $3 \times 3 \times 3$ LiH crystal. Energies are all given in Hartree.

L (Å)	Net	HF	HF correction
4.061	-8.16020(2)	-8.06092	0.0069

Supplementary Note 5. Metallic lithium

Supplementary Note 5.1 Geometry

Metallic lithium has a body-centered structure, whose lattice vectors and atom positions are given in Supplementary Table 11.

Supplementary Table 11 | Geometry of conventional bcc-Li cell

Atom	Position(Å)	lattice vector	Position(Å)
Li1	(0.0, 0.0, 0.0)	$\mathbf{a}_1$	(3.436, 0.0, 0.0)
Li2	(1.718, 1.718, 1.718)	$\mathbf{a}_2$	(0.0, 3.436, 0.0)
		$\mathbf{a}_3$	(0.0, 0.0, 3.436)

Supplementary Note 5.2 Energy results

A $2 \times 2 \times 2$ conventional cell of bcc-Li at Γ point is employed, and the calcuated results are given in Supplementary Table 12. Traditional VMC, DMC and experiment data are taken from Refs.[5, 6].

Supplementary Table 12 | Total energy and cohesive energy of bcc-Li. Twist average technique is employed to reduce the finite-size error, but we encounter an optimization problem at non-zero twist angles for bcc-Li. The cohesive energy is lifted by non-zero twist angles, and the result deviates further from the experimental data.

	Net at Γ	VMC [5]	DMC [6]	Experiment
Total energy per cell (Hartree)	-15.04180(7)	NA	NA	NA
Cohesive energy per atom (eV)	-1.168	-1.57	-1.09	-1.65

Supplementary Note 6. Homogeneous electron gas

Supplementary Note 6.1 14-electron HEG

The calculated result of closed-shell 14-electron HEG is listed in Supplementary Table. 13. FermiNet [7] and WAP-Net [8] results are listed for comparison. DMC and TC-FCIQMC results are from Refs. [9, 10], i-FCIQMC results are from Ref. [7].

Supplementary Table 13 | Energy per electron of 14-electron HEG at different mean radius of electrons r_s. Energies are all given in Hartree, r_s is given in Bohr. Electrons form a closed-shell configuration and they are restricted in a simple cubic.

r_s	Net	FermiNet-HEG	WAP-Net	BF-DMC	i-FCIQMC	TC-FCIQMC	HF
0.5	3.413258(4)	3.412683(4)	N/A	3.41370(2)	3.41226(3)	3.41241(1)	3.454894
1	0.569425(2)	0.568904(2)	0.568965(1)	0.56958(1)	0.56850(4)	0.56861(1)	0.606534
2	-0.007961(1)	-0.0084275(7)	-0.008331(3)	-0.007949(7)	-0.00873(4)	-0.00868(2)	0.023039
5	-0.0795646(3)	-0.0798213(7)	-0.079836(1)	-0.079706(3)	-0.0799(1)	-0.08002(2)	-0.058039

Supplementary Note 6.2 54-electron HEG

The training curve of closed-shell 54-electron HEG is plotted in Supplementary Fig. 7. E_{HF} and E_{DMC} are taken from Ref. [9]. Final results of neural network, BF-DMC, BF-VMC, DCD, TC-FCIQMC and HF [9–11] are listed in Supplementary Table 14.

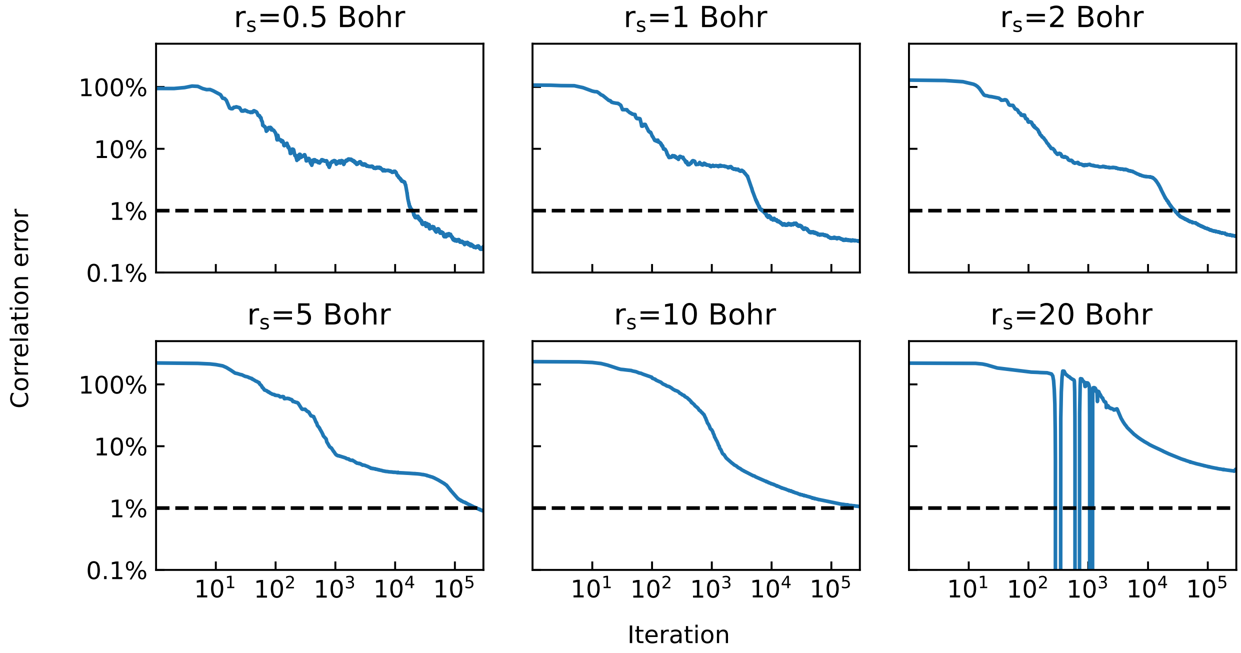

Supplementary Figure 7 | 54-electron HEG training curve. For clarity, at each iteration number, we show the median correlation error over the last 10% of iteration.

Supplementary Table 14 | Energy per electron of 54-electron HEG at different mean radius of electrons r_s. Energies are all given in Hartree, r_s is given in Bohr. Electrons form a closed-shell configuration and they are restricted in a simple cubic.

r_s	Net	BF-DMC	BF-VMC	DCD	TC-FCIQMC	HF
0.5	3.221226(2)	3.22112(4)	3.22132(7)	3.22052	3.22042	3.2659
1	0.530019(1)	0.52989(4)	0.53009(3)	0.53001	0.52973	0.5689
2	-0.013840(1)	-0.013966(9)	-0.01382(2)	-0.01286	NA	0.0186
5	-0.0788354(2)	-0.079036(3)	-0.078961(5)	-0.07655	NA	-0.05625
10	-0.0542785(1)	-0.054443(2)	-0.054389(2)	-0.05157	NA	-0.03884
20	-0.0316886(1)	-0.032047(2)	-0.0319984(8)	-0.02925	NA	-0.02205

Supplementary Note 7. Bader charge analysis

Detailed Bader charge analysis [12] is applied to conventional LiH crystal, and the result is plotted in Supplementary Fig. 8. According to the result, Li and H atoms in LiH become $Li^{0.67+}$ and $H^{0.67-}$ ions respectively.

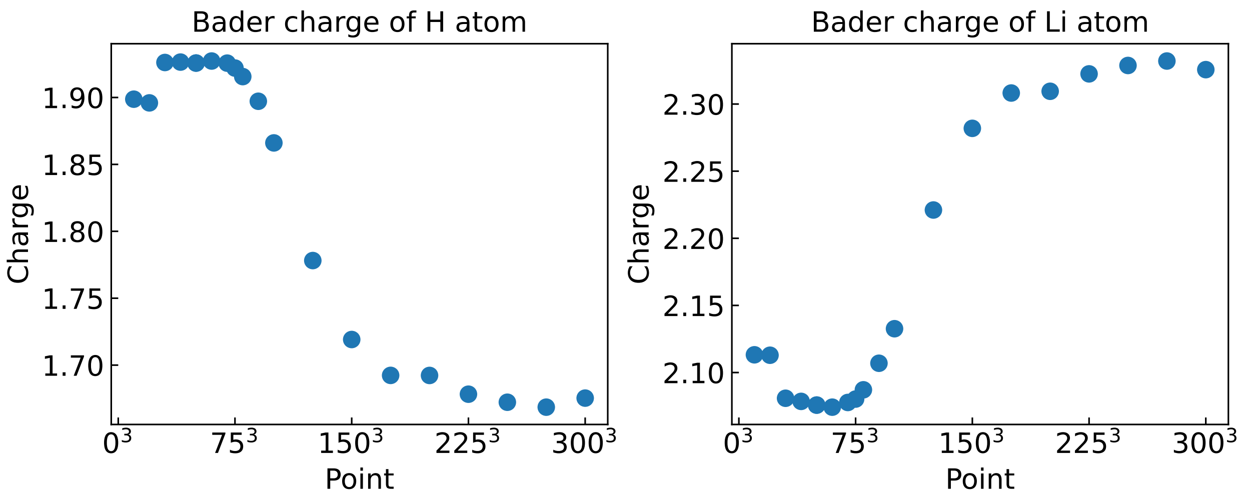

Supplementary Figure 8 | Bader charge of LiH crystal. Calculated Bader charge of atoms in LiH crystal, point denotes the number uniformly dividing the crystal.

Supplementary Note 8. Benchmark systems

A set of small systems are selected as benchmarks. Detailed geometry information is listed in Supplementary Table. 15. Recommended hyperparameters listed in Supplementary Table. 2 are employed for simulation, and the train iterations is reduced to 1e5. Calculated results are list in Supplementary Table. 16.

Supplementary Table 15 | Geometry of benchmark systems.

System	Structure	Atom	Position(Å)	lattice vector	Position(Å)
LiH	rock-salt	Li1	(0.0, 0.0, 0.0)	$\mathbf{a}_1$	(0.0, 2.0305, 2.0305)
		H	(2.0305, 2.0305, 2.0305)	$\mathbf{a}_2$	(2.0305, 0.0, 2.0305)
				$\mathbf{a}_3$	(2.0305, 2.0305, 0.0)
Li	bcc	Li1	(0.0, 0.0, 0.0)	$\mathbf{a}_1$	(3.436, 0.0, 0.0)
		Li2	(1.718, 1.718, 1.718)	$\mathbf{a}_2$	(0.0, 3.436, 0.0)
				$\mathbf{a}_3$	(0.0, 0.0, 3.436)
Be	hexagonal	Be1	(1.1299, 0.6524, 2.6774)	$\mathbf{a}_1$	(2.2598, 0.0, 0.0)
		Be2	(0.0, 1.3047, 0.8925)	$\mathbf{a}_2$	(-1.1299, 1.9571, 0.0)
				$\mathbf{a}_3$	(0.0, 0.0, 3.5699)
C	diamond	C1	(0.0, 0.0, 0.0)	$\mathbf{a}_1$	(0.0, 1.7869, 1.7869)
		C2	(0.8934, 0.8934, 0.8934)	$\mathbf{a}_2$	(1.7869, 0.0, 1.7869)
				$\mathbf{a}_3$	(1.7869, 1.7869, 0.0)
C	graphene	C1	(1.421, 0.0, 0.0)	$\mathbf{a}_1$	(2.1315, -1.2306, 0.0)
		C2	(2.842, 0.0, 0.0)	$\mathbf{a}_2$	(2.1315, 1.2306, 0.0)
				$\mathbf{a}_3$	(0, 0, 52.9177)

Supplementary Table 16 | Calculated energy of selected small systems. A $1 \times 1 \times 1$ cell at Γ point is used. HF results are calculated in cc-pVDZ basis. Energies are given in Hartree. N_e denotes number of electrons.

System	Structure	Net	HF	N_e
LiH	rock-salt	-8.5165(2)	-8.4512	4
Li	bcc	-15.34486(1)	-14.38643	6
Be	hexagonal	-30.2416(1)	-30.0752	8
C	diamond	-75.4009(2)	-74.9784	12
C	graphene	-76.0350(2)	-75.5779	12

Supplementary References

[1] Mario Motta, David M. Ceperley, Garnet Kin-Lic Chan, John A. Gomez, Emanuel Gull, Sheng Guo, Carlos A. Jiménez-Hoyos, Tran Nguyen Lan, Jia Li, Fengjie Ma, Andrew J. Millis, Nikolay V. Prokof'ev, Ushnish Ray, Gustavo E. Scuseria, Sandro Sorella, Edwin M. Stoudenmire, Qiming Sun, Igor S. Tupitsyn, Steven R. White, Dominika Zgid, and Shiwei Zhang. Towards the solution of the many-electron problem in real materials: Equation of state of the hydrogen chain with state-of-the-art many-body methods. *Phys. Rev. X*, 7:031059, Sep 2017.

[2] David Pfau, James S. Spencer, Alexander G. D. G. Matthews, and W. M. C. Foulkes. Ab initio solution of the many-electron schrödinger equation with deep neural networks. *Phys. Rev. Research*, 2:033429, Sep 2020.

[3] Simone Chiesa, David M. Ceperley, Richard M. Martin, and Markus Holzmann. Finite-size error in many-body simulations with long-range interactions. *Phys. Rev. Lett.*, 97:076404, Aug 2006.

[4] S. J. Binnie, S. J. Nolan, N. D. Drummond, D. Alfè, N. L. Allan, F. R. Manby, and M. J. Gillan. Bulk and surface energetics of crystalline lithium hydride: Benchmarks from quantum monte carlo and quantum chemistry. *Phys. Rev. B*, 82:165431, Oct 2010.

[5] G. Yao, J. G. Xu, and X. W. Wang. Pseudopotential variational quantum monte carlo approach to bcc lithium. *Phys. Rev. B*, 54:8393–8397, Sep 1996.

[6] G. Sugiyama, G. Zerah, and B.J. Alder. Ground-state properties of metallic lithium. *Physica A: Statistical Mechanics and its Applications*, 156(1):144–168, 1989.

[7] G. Cassella, H. Sutterud, S. Azadi, N. D. Drummond, D. Pfau, J. S. Spencer, and W. M. C. Foulkes. Discovering quantum phase transitions with fermionic neural networks, 2022.

[8] Max Wilson, Saverio Moroni, Markus Holzmann, Nicholas Gao, Filip Wudarski, Tejs Vegge, and Arghya Bhowmik. Wave function ansatz (but periodic) networks and the homogeneous electron gas, 2022.

[9] P. López Ríos, A. Ma, N. D. Drummond, M. D. Towler, and R. J. Needs. Inhomogeneous backflow transformations in quantum monte carlo calculations. *Phys. Rev. E*, 74:066701, Dec 2006.

[10] Hongjun Luo and Ali Alavi. Combining the transcorrelated method with full configuration interaction quantum monte carlo: Application to the homogeneous electron gas. *Journal of Chemical Theory and Computation*, 14(3):1403–1411, 2018. PMID: 29431996.

[11] Ke Liao, Thomas Schraivogel, Hongjun Luo, Daniel Kats, and Ali Alavi. Towards efficient and accurate ab initio solutions to periodic systems via transcorrelation and coupled cluster theory. *Phys. Rev. Research*, 3:033072, Jul 2021.

[12] W Tang, E Sanville, and G Henkelman. A grid-based bader analysis algorithm without lattice bias. *Journal of Physics: Condensed Matter*, 21(8):084204, jan 2009.
